# Improvement in Functional Outcomes in Patients with Benign Paroxysmal Positional Vertigo

**DOI:** 10.1155/crnm/6611253

**Published:** 2025-11-21

**Authors:** Regan G. Harrell, Susan L. Whitney

**Affiliations:** Department of Physical Therapy, The University of Pittsburgh, Pittsburgh, Pennsylvania, USA

## Abstract

This case report describes outcomes of three cases with benign paroxysmal positional vertigo (BPPV) who presented with an inability to state the symptoms of BPPV. The diagnosis is driven by patient-reported symptoms during positional testing or movement changes. People with traumatic brain injuries (TBIs) can have BPPV but report no symptoms of spinning (vestibular agnosia). The case report demonstrates that functional improvements are made in patients with vestibular agnosia. All cases were seen in an inpatient rehabilitation unit. Case 1 presented with a bilateral TBI with a daily Agitated Behavior Scale score of 41/56. She had right posterior canal BPPV yet reported no symptoms. Upon the completion of BPPV treatment, her daily Agitated Behavior Scale score decreased to 23/56. Case 2 had a multicompartment hemorrhage, with a Functional Gait Assessment (FGA) score of 11/30 before positional testing. He had right torsional upbeating nystagmus on the right Dix–Hallpike test, yet he reported no symptoms during the maneuver. After repositioning (same treatment session), his FGA improved to 19/30. Case 3 presented with a left subdural hematoma. He had left posterior canal BPPV with no symptoms during the Dix–Hallpike test. His FGA before testing was 19/30; immediately after the repositioning maneuver, his FGA was 24/30. Cases 2 and 3 met the minimally clinically important difference for the FGA of four points in the same session. People post-TBI with vestibular agnosia should be quickly treated as the canalith repositioning maneuver may reduce agitation and improve gait.

## 1. Introduction

Traumatic brain injuries (TBIs) account for more than 2.53 million emergency department visits annually [1]. TBI can cause impairments in cognition, language, perception, motor planning, balance, coordination, and safety. Deficits of TBI can be systemic and challenging to quantify given the global nature of TBI. Calzolari et al. demonstrated a phenomenon termed “vestibular agnosia,” a condition in which people with TBI have positive signs of vestibular pathology but are not able to report any symptoms related to the vestibular pathology [2]. While vestibular agnosia has been demonstrated thus far only in those with TBI, there is growing evidence that a decrease in vestibular perception occurs in other populations, including those with vascular dementia and older community-dwelling adults [3–5]. Calzolari et al. developed a vestibular perceptual laboratory test for detecting the presence of vestibular agnosia; however, this test is not yet commercially available [2].

Benign paroxysmal positional vertigo (BPPV), as defined by the American Academy of Otolaryngology-Head and Neck Surgery (AAO-HNS) Clinical Practice Guidelines, is an inner ear disorder characterized by a sudden onset of vertigo lasting 10–30 s with a delayed onset that typically lasts less than a minute and is associated with a change in head position relative to gravity [6]. While BPPV has been traditionally reported as an idiopathic condition, the rate of posttraumatic BPPV is higher than previously known [7]. Harrell et al. reported that in a TBI inpatient rehabilitation unit, the incidence of BPPV was 58% of their sample [8].

If BPPV is undiagnosed or untreated, it can lead to a reduction in activities of daily living, increased fall risk, depression, and lower quality of life [6, 9]. There is ample evidence that idiopathic BPPV when corrected influences postural control and balance. BPPV has an estimated cost of $2000 per person for unnecessary diagnostic testing and medication, and the direct and indirect healthcare costs are approaching $2 billion a year [6, 10]. While BPPV is traditionally screened for in people who report vertigo, there is growing evidence that BPPV can present without symptoms, especially in those with a traumatic mechanism of injury [3, 8]. As vestibular agnosia has only recently been described, there are limited data demonstrating that treating BPPV in this population improves function [11]. Work by Smith et al. demonstrated that those with BPPV and acute TBI had improvements in gait speed following correction of BPPV; however, not all of these subjects had vestibular agnosia and a formal balance measure was not utilized [11]. This case report demonstrates that functional outcome measures other than gait speed can improve with the correction of BPPV in those with a TBI and vestibular agnosia-like presentations.

## 2. Case Presentation

### 2.1. Case 1

Case 1 is a 75-year-old female who fell while traveling abroad. As per the patient's family, prior to the fall, she was completely independent with a past medical history including osteoporosis and hypertension. Her initial injuries included left temporoparietal, right frontal, and right temporal intracerebral hemorrhages. She also had a nondisplaced left parietal skull fracture. She was in the acute care hospital for 4 weeks prior to transferring back to inpatient rehabilitation in the United States. During her initial physical therapist examination, she had decreased attention, anxiety, impulsiveness, poor safety awareness, reduced strength, and poor sitting postural control, and she needed the following assistance for functional mobility: maximal assistance for bed mobility and maximal assistance of two people for transfers. Nursing and occupational therapy notes indicate that Case 1 demonstrated increased agitation with rolling in bed for dressing and peri-care. Her initial combination score of the GG0130 (self-care) and GG0170 (mobility) of the Inpatient Rehabilitation Facility Patient Assessment Instrument (IRF-PAI) was 19. Her initial Orientation Log was not able to be assessed secondary to poor cognition. Orientation Log is a quantitative measure of orientational status. Place, time, and situational domains are assessed. Scores of > 25/30 are associated with normal orientation [12]. Given that Case 1 was unable to complete the Orientation Log, it can be inferred that her cognitive status was severely impaired as she was not aware of who she was, where she was, and what the time of day was. Case 1 could express the words “yes” and “no,” but as per a Speech Language Pathology note, “[Case 1] was able to answer yes/no questions by articulating yes/no, she was accurate 1/5 times when asked if her name was [Case 1] and 0/5 times if she was in a hospital.” These results are shown in Table 1.

Another outcome used with Case 1 was the Agitated Behavior Scale (ABS). The ABS is a 14-item instrument that can identify three forms of agitation in a person with a TBI: disinhibition, aggression, and lability [13]. The items are scored on a one to four scale, with one indicating that the behavior is absent and four indicating that the behavior exists and is interfering with function [13]. The ABS has been demonstrated as valid and reliable in assessing agitation, and a standard nursing practice is that anyone with an ABS of over 20 should have constant supervision for safety [14].

Secondary to agitation, poor cognition, and fluctuating arousal, Case 1's BPPV assessment was completed on Day 14 of her inpatient rehabilitation stay. Before positional testing, she was asked the following questions based on work by Kim et al., “Do you experience dizziness when you get out of bed?” and “Do you experience dizziness when you roll over in bed?” [15]. Whitney et al. reported that when questions regarding bed mobility were answered “yes,” there was a 4.3 times increased likelihood of the person having BPPV [16]. Case 1 answered “yes” to all questions; however, Case 1 also answered “yes” to “Do you get dizzy when you go from sitting to standing?” and at this point in her plan of care, Case 1 had stood only once during her initial examination. Case 1 had left torsional upbeating nystagmus during the Dix–Hallpike test, indicating left posterior canal BPPV. Three canalith repositioning maneuvers (CRMs) were performed, and a negative Dix–Hallpike test was achieved upon the third maneuver. Throughout the testing and CRM, Case 1 had no symptoms of dizziness or vertigo, nor did she report lightheadedness or imbalance during the maneuvers. In the following 24 h post-CRM, her ABS dropped 18 points from 41/56 to 23/56. Her outcomes post-CRM are shown in Table 2.

### 2.2. Case 2

Case 2 was a 40-year-old male who, after a bicycle accident, presented to the emergency department with a left wrist fracture, left temporal bone fracture, and facial and head lacerations. Imaging confirmed that Case 2 had a multicompartment acute intracranial hemorrhage (right temporal subdural hematoma, left subdural hematoma, bilateral frontal contusions, left temporal parenchymal contusions, and a left anterior Sylvian fissure subarachnoid hemorrhage) as reported by the radiology report from the computed tomography scan without contrast. He spent 2 days in acute care before being admitted to inpatient rehabilitation. During his initial examination, Case 2 required moderate assistance for bed mobility, transfers, and gait of 50 feet. Case 2's initial IRF-PAI and Orientation Log scores are shown in Table 1. His impairments included decreased attention, poor safety awareness, and restlessness, and he was able to follow one-step commands with 25% accuracy, and his Functional Gait Assessment (FGA) was 0/30. The FGA is a walking test that assesses dynamic balance during gait, with scores of < 23/30 indicating an increased risk of falling [17, 18].

On his fifth day of inpatient rehabilitation, he required minimal assistance for ambulation, followed simple commands 100% of the time, and was restless. His FGA on Day 5 was 11/30. On Case 2's sixth day of inpatient rehabilitation, he was assessed for BPPV. He answered “no” to both screening questions. Case 2 had typical right posterior canal BPPV and was treated with two CRMs, resulting in a negative Dix–Hallpike test afterward. He was not symptomatic during positional testing or during the modified Epley intervention. His outcomes are shown in Table 1. Immediately following the CRM, his FGA score was 19/30.

### 2.3. Case 3

Case 3 was a 65-year-old male who presented to the emergency department after a fall down stairs. He experienced a left subdural hematoma with a midline shift. He was in acute care for 6 days before being admitted to inpatient rehabilitation. Upon initial evaluation, he was alert and oriented to self and place, lethargic, and demonstrated impulsivity. His initial IRF-PAI score and Orientation Log are shown in Table 1. He required minimal assistance for mobility, his gait speed was 0.55 m/s, and his FGA was 0/30. After 10 days of inpatient rehabilitation, he was oriented to self and place, his impulsivity had not improved, his FGA was 19/30, and his gait speed was 1.38 m/s. He was assessed for BPPV on Day 12 of his inpatient rehabilitation stay. He responded “no” to the BPPV screening questions. He had left posterior canal BPPV and was treated with two CRMs, with a negative Dix–Hallpike test after the CRMs. He reported no symptoms during both the positional testing and CRMs. Immediately following the CRM intervention, his FGA score was reassessed and was 24/30. His outcomes are shown in Table 2.

## 3. Results

All three cases experienced resolution of their BPPV using the CRM. Case 1's ABS score decreased from 41 to 23 within 24 h post-CRM, which was not a minimally clinically important difference. However, nursing and occupational therapy notes indicate that as there was less agitation during bed mobility, she required the assistance of only one person for dressing and transfers following the CRM. Clinically important improvements in the FGA for Cases 2 and 3 following the CRM are shown in Table 2.

## 4. Discussion

Cases 1, 2, and 3 did not report symptoms consistent with BPPV but had the classic torsional nystagmus that is diagnostic of BPPV. Previous work by Harrell et al. demonstrated that the incidence of BPPV in a population of people with TBI in an inpatient rehabilitation setting was 58%, with only 10% of those who tested positive able to report any symptom associated with BPPV [8]. Calzolari's work was the first to test for vestibular agnosia using a standardized laboratory test created by Dr. Seemungal to test the perception in all their subjects [2]. The cases in this study did not complete this laboratory test as the Calzolari perception test is not commercially available. While it is impossible to identify with certainty that these three cases have vestibular agnosia, they present with a clinical presentation that matches someone with a lack of perception of vestibular sensation.

While the underlying mechanisms of vestibular agnosia are not completely understood, it has been shown that it is more than just damage to one area in the cortex [2, 19, 20]. Vestibular agnosia has been identified primarily in those with TBI, and there have also been reported cases in those with vascular dementia and spinocerebellar degeneration [20]. In Hadi's study, they discovered that changes in the gray matter in the left hemisphere including the supplementary motor areas, precuneus, mid-frontal gyrus, and paracentral lobule had significant interactions with behavioral measures [19]. In those who recovered from their vestibular agnosia, there was increased connectivity in the splenium, forceps major, and body of the corpus callosum, indicating that while damage to the left hemisphere might be responsible for the initial lack of perception, communication between the hemispheres may be required in recovery [19]. While all three cases in this case report had different mechanisms of TBI and locations of injury, all three had initial damage to the left hemisphere including the parietal lobe.

Given the complex nature of TBI, other causes of the nystagmus were ruled out including central positional nystagmus (CPN). CPN is thought to be from an impaired integration of peripheral vestibular information within the brainstem and cerebellum [21, 22]. In those with CPN, there is an atypical nystagmus pattern during positional testing that fails to resolve with treatment [22, 23]. While all three cases in this case report had damage to bilateral hemispheres, there were no imaging data suggesting damage to the cerebellum or brainstem. All three cases also had a consistent nystagmus pattern with posterior canal BPPV, and all three responded to treatment as seen with negative positional testing after treatment.

A recent systematic review by Pauwels et al. demonstrated a significant improvement in balance, as measured by the FGA or the Dynamic Gait Index (DGI), in those with idiopathic BPPV [24]. This case report is the first to demonstrate the same improvement in functional balance measures in those with vestibular agnosia. While balance deficits are a common impairment following TBI, both Cases 2 and 3 improved their FGA scores by 8 and 5, respectively. These improvement scores meet the minimally clinically important difference of four points for the FGA [25]. Case 3's final FGA score, 24/30, also improved to over the fall risk cutoff score of greater than 22/30 [17]. The FGA score for both cases was assessed the day before their BPPV assessment and treatment and then repeated within the same treatment session of the CRM. Given that there was only a day between assessments, and the only change in the plan of care that both cases experienced was treatment of their BPPV, it appears that correcting their BPPV, even when the person does not report symptoms of dizziness, improved their balance and reduced their fall risk.

Agitation is a broad term for a state of behavioral excess, including aggression, restlessness, disinhibition, and/or lability [26]. While agitation as an umbrella term can vary greatly from person to person postinjury, up to 36% of people post-TBI will experience some form of agitation [27]. Work from Bogner et al. demonstrates that people with higher levels of agitation at admission to inpatient rehabilitation were more likely to have longer lengths of stay, lower cognitive functioning at discharge, and a decreased likelihood that they would be discharged to a private residence [28]. There is no known minimally clinically important difference for the ABS, and scores of 21 or below indicate normal functioning, scores of 22–28 indicate mild agitation, scores of 29–35 indicate moderate agitation, and scores greater than 35 indicate severe agitation [28]. Case 1's ABS score before BPPV treatment was 41, indicating moderate agitation. After treatment, her ABS score was 23, indicating mild agitation. While it is unknown if this is clinically significant, the improvement in Case 1's agitation decreased the burden of care she required during inpatient rehabilitation.

Harrington et al. state that in skilled nursing facilities in the United States, up to 54% of skilled nursing homes did not meet the total Centers for Medicare & Medicaid Services expected staffing level 80% of the time [29]. Based on Case 1 needing two people for transfers and bed mobility before her BPPV treatment, she would have been in the highest complexity patient—extensive services—in the Resource Utilization Group (RUG) and, by CMS standards, would need a total of 7.68 h of nursing staff daily. After her BPPV treatment and decrease in agitation, her RUG score would be in the special care low category, requiring 5.79 total hours of nursing staff daily. It appears that the resolution of her BPPV may have influenced her agitation. A reduction in agitation can lead to a lower burden of care in both inpatient rehabilitation and skilled nursing facilities.

This case report is not without limitations. There is no definitive way to know if these cases had true vestibular agnosia as defined by Calzolari's group or if their inability to communicate any perceived symptoms was related to cognitive impairment. The current test for vestibular perception is reliant on the person being able to understand the components of the test and then respond appropriately [2]. It is well understood that after TBI, a wide range of cognitive problems can present [30, 31]. Any number of these cognitive impairments can hinder the person from feeling, expressing, or understanding the feelings of vertigo. In a study of physical therapists' responses to whether they would screen someone for BPPV based on symptom complaints, 62% of respondents would always assess someone for BPPV when presented with classic dizziness with head movement complaints [32]. In the same paper, when presented with a case of someone without complaints of vertigo, the rate of assessment for BPPV decreased to less than 40% [32]. Based on current practice, these three cases would not have been screened for BPPV as they did not report symptoms. The improvements in outcome measures demonstrate that even if there are no symptoms, there are still physiological effects. These cases represent three individuals who traditionally would not have been assessed for BPPV but had BPPV and showed functional improvements. Still, the cases provide a good starting point for further, more extensive prospective studies.

## Figures and Tables

**Table 1:** Demographics of Cases 1, 2, and 3.

	Case 1	Case 2	Case 3
Age (gender)	75 (F)	40 (M)	64 (M)
Initial Orientation Log^∗^	Unable to assess	14	24
Initial combination score of the GG0130 (self-care) and GG0170 (mobility) of the Inpatient Rehabilitation Facility Patient Assessment Instrument (IRF-PAI)^∗∗^	19	25	54
BPPV screening items			
Do you experience a room-spinning sensation when you get out of bed?^∗∗∗^	Yes	No	No
Do you experience a room-spinning sensation when you roll over in bed?^∗∗∗^	Yes	No	No
Dix–Hallpike	Positive (left posterior canal)	Positive (right posterior canal)	Positive (left posterior canal)
Roll test	Not tested	Negative	Negative

^∗^Orientation Log is a quantitative measure of orientational status. Place, time, and situational domains are assessed. Scores of > 25/30 are associated with normal orientation.

^∗∗^Inpatient Rehabilitation Facility Patient Assessment Instrument is the instrument that Inpatient Rehabilitation Facilities use to collect patient assessment data for payment determination. It is required for all patients. Lower scores indicate that a person requires more assistance for daily activities.

^∗∗∗^Questions adapted from Kim et al. and Whitney et al. work using the Dizziness Handicap Index to identify benign paroxysmal positional vertigo (BPPV) (16, 17).

**Table 2:** Outcomes pre- and post-canalith repositioning maneuvers (CRMs).

Patient	Outcome	Pre-CRM (within the previous 24 h of CRM)	Post-CRM (within 24 h post CRM)	Clinical utility
Case 1	Agitated Behavior Scale	41	23	Went from moderate agitation to mild agitation [14]
Case 2	Functional Gait Assessment	11/30	19/30	Minimally clinically important difference is 4 points [25]
Case 3	Functional Gait Assessment	19/30	24/30	Minimally clinically important difference is 4 points [25] Increased risk of fall with scores < 22/30 [17]
